# Tuberculosis among Foreign-born Persons, Singapore, 2000–2009

**DOI:** 10.3201/eid1703.101615

**Published:** 2011-03

**Authors:** Khin Mar Kyi Win, Cynthia B.E. Chee, Liang Shen, Yee T. Wang, Jeffery Cutter

**Affiliations:** Author affiliations: Tan Tock Seng Hospital, Singapore (K.M. Kyi Win, C.B.E. Chee, Y.T. Wang);; National University of Singapore, Singapore (L. Shen);; Ministry of Health, Singapore (J. Cutter)

**Keywords:** Epidemiology, tuberculosis, tuberculosis and other mycobacteria, bacteria, foreign-born persons, Singapore, dispatch

## Abstract

We determined the proportion of foreign-born persons with tuberculosis (TB) in Singapore. This proportion increased from 25.5% in 2004 to 37.6% in 2009. Unskilled workers from countries with high incidences of TB accounted for the highest number of and greatest increase in foreign-born TB case-patients.

Singapore, an island city-state (area 710 km^2^) in Southeast Asia, liberalized its immigration policy and underwent rapid economic growth during 2005–2010. This policy resulted in a marked increase in its population from 4.17 million in 2004 to 5.08 million in 2010, which was largely caused by an increase in foreign-born persons comprising long-term pass holders (LTPHs) (permission to stay in Singapore >6 months), permanent residents (PRs), and naturalized citizens (*1*). In recent decades, mass immigration and influx of nonimmigrants from countries with high incidences of tuberculosis (TB) to industrialized countries have contributed to the epidemiology and incidence of TB in host countries (*2**–**9*). We report the epidemiology of TB in foreign-born case-patients in Singapore during 2000–2009.

## The Study

Notification of TB cases to the Singapore TB Elimination Programme (STEP) Registry is mandated by law. Since 1998, the notification report has included disease characteristics, sociodemographic information, country of origin, immigration status, and year of arrival in Singapore of TB patients (*10*). All notifications during 2000–2009 were obtained from the STEP Registry database. When data on country of birth were not available for persons not born in Singapore (0.5% of case-patients), the nationality of these persons was assumed to represent their country of birth. Missing information about country of birth for persons with a Singaporean nationality (17%) and arrival date (6.5%) were matched against a database provided by the Ministry of Home Affairs.

Persons not born in Singapore were considered foreign born. Foreign-born case-patients comprised naturalized citizens, PRs, and LTPHs. Singapore issues long-term passes to skilled and unskilled workers and others (students and foreign-born family members of citizens or PRs) to enable a stay >6 months in Singapore. Persons applying for permanent residency or long-term stay and renewal of long-term passes are given medical examinations that include a general physical examination, chest radiograph, and testing for HIV (*11*). The Department of Singapore Statistics provided 2000–2010 population data. We estimated the number of persons born in Singapore during 2000–2010 by using linear interpolation based on 2000 and 2010 population census exercises. Consistent with the country classification used in the Singapore 2000 population census, People’s Republic of China, Hong Kong, and Taiwan were classified as 1 group of country of birth.

**Figure 1:**
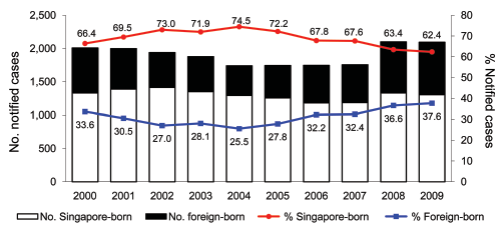
Tuberculosis (TB) cases and proportion of native-born versus foreign-born persons, Singapore, 2000–2009. Numbers along data lines indicate percentage of native-born persons with TB versus foreign-born persons with TB.

There were 23,164 cases of TB (new and re-treated) reported to the registry during 2000–2009. After we excluded 4,033 short-term pass holders, persons whose TB diagnosis had changed (n = 97), and persons with missing information about country of origin (n = 34), a total of 19,000 persons were eligible for the study. Of these persons, 13,048 (68.7%) were born in Singapore and 5,952 (31.3%) were foreign born. The number and proportion of foreign-born cases decreased in the first half of the study period from 675 (33.6%) in 2000 to 444 (25.5%) in 2004. This trend reversed during 2005, and the number and proportion of foreign-born persons with TB increased to 788 (37.6%) in 2009 (Figure 1). Of foreign-born persons with TB, 3,386 (56.9%) were LTPHs, 1,820 (30.6%) were PRs, and 746 (12.5%) were naturalized citizens.

**Figure 2:**
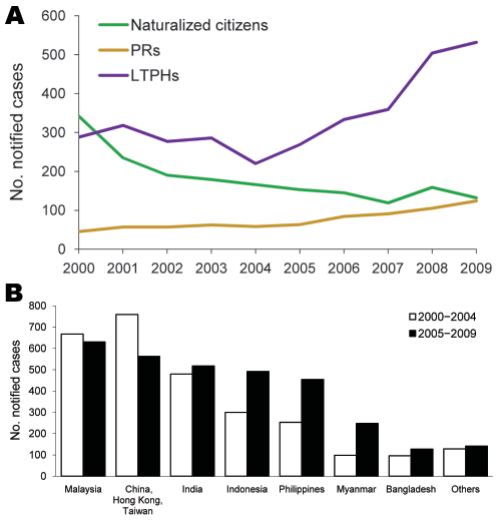
Tuberculosis (TB) cases, Singapore, 2000–2009. A) No. notified cases among foreign-born subgroups, by year of notification. Citizens, naturalized citizens; PRs, permanent residents; LTPHs, long-term pass holders. B) No. notified cases by country of origin.

LTPHs with TB comprised 2,562 (75.7%) unskilled workers, 371 (10.9%) professional and skilled workers, and 162 (4.8%) students. The remaining 291(8.6%) persons were foreign-born family members of Singapore citizens or PRs who were granted long-term stay in Singapore. TB among LTPHs increased from 220 (49.5%) cases in 2004 to 532 (67.5%) in 2009 and that among PRs increased from 45 (6.7%) in 2000 to 124 (15.7%); cases among foreign-born citizens decreased from 342 (50.7%) in 2000 to 132 (16.8%) in 2009 (Figure 2, panel A).

The number of persons with TB from Indonesia, the Philippines, and Myanmar increased from 2000–2004 to 2005–2009 (Figure 2, panel B). Among native-born persons, TB occurred predominantly in men >40 years of age (Table). TB in foreign-born persons was distributed equally in both sexes and occurred mostly in persons 20–39 years of age (Table). Drug resistance and extrapulmonary involvement were higher among foreign-born persons than among native-born persons.

**Table:** Characteristics of native-born and foreign-born persons with TB, Singapore, 2000–2009*

Characteristic	No. (%) native-born, n = 13,048	No. (%) foreign-born, n = 5,952
Sex		
F	3,546 (27.2)	2,731 (45.9)
M	9,502 (72.8)	3,221 (54.1)
Age, y		
Median	53.2	33.8
0–19	516 (4.0)	149 (2.5)
20–39	2,592 (19.9)	3,503 (58.9)
40–59	5,137 (39.4)	812 (13.6)
>60	4,803 (36.8)	1,488 (25.0)
Diabetes		
No	10,282 (78.8)	5,409 (90.9)
Yes	2,766 (21.2)	543 (9.1)
New case	11,550 (88.5)	5,588 (93.9)
Re-treated case	1,498 (11.5)	364 (6.1)
Site of disease		
Pulmonary†	11,447 (87.7)	4,832 (81.2)
Extrapulmonary	1,601 (12.3)	1,120 (18.8)
Site of extrapulmonary TB‡		
Lymph node	571 (35.7)	582 (52.0)
Pleura	415 (25.9)	258 (23.0)
Other	698 (43.6)	328 (29.3)
Sputum smear for AFB		
Negative	5,744 (44.0)	2,790 (46.9)
Positive	4,784 (36.7)	1,532 (25.7)
Not determined	2,520 (19.3)	1,630 (27.4)
Sputum culture for MTC		
Negative	3,075 (23.6)	1,819 (30.6)
Positive	8,316 (63.7)	2,855 (48.0)
Not determined	1,657 (12.7)	1,278 (21.5)
Drug resistance		
MDR§	28 (0.3)	39 (1.4)
Isoniazid¶	256 (3.1)	226 (7.9)

*TB, tuberculosis; AFB, acid-fast bacilli; MTC, *Mycobacterium tuberculosis* complex; MDR, multidrug resistant. †Includes pulmonary TB cases with extrapulmonary involvement. ‡Pure extrapulmonary and not mutually exclusive. §Resistant to at least isoniazid and rifampin. ¶Includes MDR; resistant to isoniazid alone or isoniazid in combination with any other first-line drugs.

## Conclusions

The decreasing trend of foreign-born persons with TB in the first half of the study period could be attributed to fewer transient migrant workers entering Singapore during the economic crisis in Asia and the severe acute respiratory syndrome outbreak in 2003. After economic recovery and liberalization of the immigration policy in Singapore in 2005, there was an influx of migrant workers and immigrants from countries with high incidences of TB and a corresponding increase in TB notifications among this population. Of these persons with TB, >75% came from 5 of the 7 countries (India, China, Indonesia, Bangladesh, and the Philippines) with highest incidences of TB (*12**,**13*).

The large increase in the number of TB case-patients from Indonesia, the Philippines, and Myanmar reflects the influx of migrant workers from these countries. This finding is consistent with the predominance of cases reported in foreign-born persons 20–39 years of age. The lower incidence of diabetes among these younger persons than in native-born persons is not surprising. The preponderance of lymph node involvement among the foreign-born persons merits further study.

The estimated TB rate in persons born in Singapore decreased from 49.7 cases/100,000 population to 41.8/100,000 during 2000–2007 but increased to 46.5/100,000 in 2008 and 45.2/100,000 in 2009. This increase followed the increase in cases among foreign-born persons in 2005. Transmission from foreign-born to native-born persons may have occurred, especially in increasingly crowded urban settings of Singapore. However, this proposal cannot be verified without DNA fingerprinting studies.

Currently, persons with untreated inactive lesions or scarring on their screening chest radiographs are given permission for a long-term stay in Singapore without further examination or follow-up. To improve screening, mandating sputum TB cultures may be prudent for these persons. If active disease is excluded, these persons can then receive prophylactic treatment.

We do not favor a policy of screening for latent TB infection because of the high probability of false-positive tuberculin skin test results in persons vaccinated with *Mycobacterium bovis* BCG and the unfavorable risk-benefit ratio of preventive therapy for persons from countries with high TB incidences, who may be more likely to have acquired latent TB in the past than recently (*14*). Although interferon-γ release assays will overcome the problem of false-positive tuberculin skin test results in persons vaccinated with *M*. *bovis* BCG, the cost of these assays is prohibitively high, and their positive predictive value for progression to active TB remains to be determined (*15*).

A strength of this study was its nationally representative data of all TB cases reported to the STEP Registry. Electronic linkage of the Registry to the 2 mycobacterial culture laboratories in Singapore also enabled inclusion of all bacteriologically positive cases. A limitation of this study was the unavailability of data for native-born and foreign-born persons by sex and age to compare age- and sex-specific TB case rates between these 2 groups. In addition, because of the lack of population data for country of birth, we were unable to determine the TB rate by country of origin. Also, information bias is inherent in retrospective studies, and most country of birth data were based on information submitted by the notifying physician. We were also unable to analyze the association of HIV and TB because access to HIV status in our study population was limited. Our study highlights the need for measures to address TB among foreign-born persons in Singapore.
